# Interpreting the Evidence on Wine Consumption and Health: A Narrative Review of the J-Shaped Association and Contemporary Alcohol Guidelines

**DOI:** 10.3390/nu18172752

**Published:** 2026-08-22

**Authors:** Creina S. Stockley, Robert Curtis Ellison, Mladen Boban

**Affiliations:** 1 School of Agriculture, Food and Wine, Adelaide University, Waite Campus, Glen Osmond, SA 5064, Australia; 2 Boston University Chobanian & Avedisian School of Medicine (formerly Boston University School of Medicine), Boston, MA 02118, USA; ellison@bu.edu; 3 Department of Basic and Clinical Pharmacology, University of Split School of Medicine, Šoltanska 2A, 21000 Split, Croatia

**Keywords:** wine, alcohol consumption, polyphenols, health, mortality, J-shaped

## Abstract

Moderate wine consumption has historically been associated with a J-shaped relationship between dose and total mortality, driven primarily by cardiovascular protection, enhanced endothelial function, improved glucose metabolism, and anti-inflammatory and antioxidant effects attributable to both ethanol and grape-derived phenolic constituents. Yet in recent years, international alcohol guidelines have shifted markedly towards the assertion that ‘no safe level’ of alcohol exists. This transition has occurred despite the continued consistency of mechanistic, clinical, and epidemiological evidence demonstrating hormetic (biphasic) responses to wine and its constituents and despite methodological controversies over the mathematical models used in several recent national guideline revisions. This narrative review synthesises mechanistic evidence on hormesis and wine-specific biological effects—including polyphenols, resveratrol, nitric oxide signalling, platelet modulation, and endothelial benefits—with epidemiological data on J-shaped associations with cardiovascular disease, diabetes, and total mortality. It further examines key methodological changes in recent guidelines, particularly the adoption of the ‘Sheffield model’ and the exclusion of or reduced emphasis on cardioprotective outcomes. The review argues that these methodological decisions, rather than new scientific evidence, largely explain the departure from earlier guideline thresholds that allowed moderate wine consumption. Understanding wine’s biological specificity, the differential health implications of drinking patterns and contexts, and the limitations of model-driven policy changes is essential to develop coherent, evidence-informed public health recommendations.

## 1. Introduction

The relationship between alcohol consumption and health has long been central to epidemiology, nutritional science, and public health discourse. Among alcoholic beverages, wine has attracted particular attention for its complex matrix of grape-derived phenolic compounds with antioxidant and anti-inflammatory properties, as well as for its cardiometabolic effects that extend beyond those attributable to ethanol alone. Converging evidence from mechanistic, experimental, and epidemiological studies has consistently supported a J-shaped association between wine consumption and total mortality, with light-to-moderate intake linked to a lower mortality risk than both abstaining and heavy drinking. This effect is driven predominantly by reductions in cardiovascular disease (CVD), alongside favourable metabolic and endothelial outcomes [1].

Over the past decade, however, public health guidance in several countries, including Australia, Canada, and the United Kingdom (U.K.), has shifted towards more restrictive recommendations. Contemporary guidelines increasingly adopt linear risk models, asserting that any level of alcohol consumption increases health risk and that no safe threshold exists. These positions diverge from earlier frameworks, which differentiated risk by beverage type, drinking pattern, and dose and acknowledged cardioprotective associations with moderate wine consumption.

Despite the large number of reviews examining alcohol consumption and health, an important knowledge gap remains in interpreting evidence specifically relating to wine. Most contemporary reviews treat alcohol primarily as a source of ethanol, combining wine, beer, and spirits into a single exposure expressed as grams of alcohol. While appropriate for estimating ethanol-attributable risk, this approach cannot determine whether associations observed for wine differ from those associated with alcohol consumption more generally. Moreover, it does not distinguish the potential contributions of wine’s non-alcoholic constituents, drinking with meals, Mediterranean dietary patterns, and other lifestyle factors that frequently accompany wine consumption.

At the same time, recent methodological developments, including greater recognition of former-drinker bias and residual confounding, the use of Mendelian randomisation, and burden-of-disease modelling, have fundamentally changed how observational evidence is interpreted. Although these approaches have challenged causal interpretations of the J-shaped association, they have rarely been considered alongside beverage-specific epidemiological evidence, mechanistic studies, and intervention trials on wine. Consequently, the central unresolved question is no longer whether a J-shaped association has been reported but how to interpret wine-specific evidence in light of contemporary methodological advances.

The J-shaped relationship between alcohol consumption and health outcomes is supported by a long-standing, reproducible body of evidence. Early observations by Pearl (1926) reported lower mortality among moderate drinkers than among abstainers and heavy drinkers [2]. Subsequent cohort studies, including the Framingham Heart Study [3] and the Kaiser Permanente cohort [4,5], confirmed a reduced risk of coronary heart disease among moderate drinkers. This pattern has been consistently replicated across diverse populations and settings, including Honolulu, Whitehall, Puerto Rico, Busselton, and North Karelia, as well as in large meta-analyses of all-cause mortality [6,7,8]. The consistency of these findings across populations supports the view that the J-shaped association is a reproducible and robust epidemiological pattern rather than an isolated finding.

Importantly, the principal change over recent decades has not been the emergence of fundamentally different biological or epidemiological evidence but rather changes in how that evidence is interpreted and how population risk is estimated. The divergence in contemporary guidance reflects evolving methodological approaches to risk estimation. Recent guideline revisions often rely on modelling frameworks that exclude cardioprotective outcomes, omit total mortality, or treat alcohol as a uniform exposure, thereby failing to capture beverage-specific effects [9]. These approaches can substantially alter risk estimates and lead to more restrictive recommendations, even when the underlying science has not changed.

The biological plausibility of wine-specific effects is supported by multiple convergent mechanisms. Moderate wine consumption (approximately 10–20 g of ethanol per day) has been associated with increased high-density lipoprotein cholesterol, reduced platelet aggregation, enhanced fibrinolysis, improved endothelial function, and reduced oxidative stress, effects attributable to both ethanol and wine-derived phenolic compounds [10,11]. *In vitro*, animal, and human studies support these mechanisms, which align with epidemiological evidence indicating a reduced risk of coronary heart disease, type 2 diabetes, and cognitive decline among moderate wine consumers [12,13,14].

Despite this, recent guideline revisions have placed greater emphasis on alcohol-related cancer risk, often without adequately accounting for modifying factors such as drinking pattern, beverage type, or dietary context. In addition, some modelling approaches assume additive, age-independent risk, which may overestimate harm at low levels of intake and fail to account for competing risks, particularly the dominant contribution of CVD to mortality in older populations. Although analyses such as the Global Burden of Disease study recognise age-dependent effects, including lower mortality among older adults with lower consumption levels, this nuance is frequently absent from public health messaging.

The shift towards universal ‘no safe level’ messaging also raises questions about its real-world effectiveness. Evidence assessing the impact of revised drinking guidelines suggests that although public awareness of the revisions may increase, there is currently limited evidence that introducing new guideline thresholds alone yields substantial or sustained reductions in alcohol consumption [15,16]. Broader long-term declines in alcohol consumption observed in several countries are likely to reflect multiple interacting influences, including changing social norms, demographic shifts, pricing policies, health awareness, and other public health interventions, rather than the guideline revisions themselves.

These considerations highlight the central challenge that this review addresses: interpreting wine-specific associations by integrating beverage-specific epidemiology with mechanistic, intervention, Mendelian randomisation, and contemporary risk-modelling evidence, while accounting for drinking patterns, wine composition, and residual confounding. While higher levels of consumption are clearly harmful, particularly for cancer and liver disease [17], CVD remains the leading cause of mortality globally, and moderate wine consumption may confer a net benefit in certain populations.

Throughout this review, “moderate” wine consumption broadly refers to intake levels at the nadir of the J-shaped association reported in prospective cohort studies. Although the precise threshold varies by study population, sex, health outcome, drinking pattern, and method of exposure assessment, the lowest observed risk has generally been reported at average intakes of approximately 10–30 g ethanol/day, with many cardiovascular outcomes showing the greatest benefit at around 10–20 g/day [6,7,9]. Because definitions of a standard drink differ internationally, alcohol intake is expressed primarily in grams of ethanol wherever possible.

Accordingly, this narrative review aims not only to summarise the evidence on wine and health but also to critically assess how the wine literature should be interpreted in light of contemporary methodological advances and evolving public health guidance. By integrating mechanistic evidence, intervention studies, beverage-specific epidemiology, Mendelian randomisation, and policy approaches within a single framework, this review seeks to clarify what can and cannot be concluded about the relationship between moderate wine consumption and health.

Specifically, it:

(1) summarises biological and mechanistic evidence supporting wine-specific biological effects and hormesis;
(2) critically evaluates beverage-specific epidemiological evidence for J-shaped associations across key health outcomes;
(3) examines how contemporary methodological approaches, including risk modelling and Mendelian randomisation, have shaped the interpretation of the evidence and the basis for alcohol guideline development; and
(4) proposes an integrated framework for interpreting the evidence and discusses implications for nutrition science, public health policy, and consumer-facing recommendations.

## 2. Review Methodology

This narrative review was prepared in accordance with the Scale for the Assessment of Narrative Review Articles (SANRA), which provides recommendations to improve the transparency and methodological quality of narrative reviews. Although a formal systematic review or meta-analysis was not undertaken, a structured literature search and a transparent study selection process were used to identify, appraise, and synthesise evidence across mechanistic, experimental, clinical, epidemiological, and policy domains. The review synthesises evidence on the health effects of wine consumption, with particular emphasis on the biological basis of the reported J-shaped relationship between wine intake and health outcomes, and critically examines the methodological foundations of recent international alcohol guidelines.

### 2.1. Search Strategy and Evidence Sources

Electronic searches were conducted in PubMed/MEDLINE, Scopus, and Web of Science. The searches were undertaken between January and March 2026 and covered publications from January 1980 to March 2026. Earlier landmark studies considered foundational to the field (e.g., Pearl, 1926 [2]) were also included where relevant to the historical development of the J-shaped hypothesis.

A structured yet flexible approach was used to identify the relevant literature. PubMed, Scopus, and Web of Science were searched using the following terms:

- “wine” AND “mortality”
- “wine” AND “cardiovascular”
- “wine polyphenols”
- “resveratrol” AND “hormesis”
- “alcohol guidelines” AND “methodology”
- “J-shaped curve” AND “epidemiology”
- “alcohol modelling” OR “Sheffield model”
- “alcohol consumption” AND “cancer risk”

Reference lists of key systematic reviews, meta-analyses, and seminal publications were manually searched (snowballing) to identify additional relevant studies. Only peer-reviewed English-language publications were included.

### 2.2. Inclusion and Exclusion Criteria

Inclusion criteria: The inclusion and exclusion criteria were defined using a PICOS framework and are summarised in Table 1. 

Exclusion criteria:

- studies without separable wine data where beverage-specific evidence was central to the review;
- conference abstracts and unpublished reports;
- narrative opinion articles without supporting evidence;
- single case reports; and
- studies concerned exclusively with heavy or binge drinking that did not inform questions relating to moderate wine consumption.

### 2.3. Integration of Mechanistic and Epidemiological Evidence

Because the biological effects of wine cannot be attributed solely to ethanol, mechanistic evidence on phenolic compounds, oxidative stress, inflammation, endothelial function, and metabolic signalling was considered alongside epidemiological and clinical evidence. Rather than relying on any single study design, evidence was synthesised from complementary research approaches, including mechanistic investigations, human intervention studies, prospective cohort studies, systematic reviews, meta-analyses, Mendelian randomisation studies, and guideline modelling exercises. Greater weight was given to evidence demonstrating consistency across multiple study designs and biological plausibility.

### 2.4. Narrative Synthesis and Assessment of Evidence

The review intentionally included evidence reporting beneficial associations, null findings, and adverse health outcomes. Studies were selected not for whether they supported a particular hypothesis but for their relevance, methodological quality, influence within the field, and contribution to understanding wine-specific effects or contemporary guideline development. Particular attention was paid to studies examining potential sources of bias, including former-drinker bias, residual confounding, reverse causation, socioeconomic confounding, beverage misclassification, and the assumptions underpinning comparative risk modelling. Evidence from observational epidemiology, intervention studies, mechanistic research, Mendelian randomisation analyses, and burden-of-disease models was synthesised to provide a balanced interpretation of the available evidence. The narrative synthesis drew on the complementary contributions of these evidence streams while highlighting key methodological limitations, sources of uncertainty, and areas where findings converge or diverge.

### 2.5. Approach to Guideline Analysis

Guideline documents from Australia (2020), the U.K. (2016), Canada (2023), and the Netherlands (2015) [18,19,20,21] were examined, with attention to:

- primary evidence sources cited;
- modelling assumptions;
- inclusion or exclusion of cardioprotective endpoints;
- treatment of beverage-specific effects; and
- risk thresholds and acceptable risk definitions.

## 3. Results

The results are presented in six major subsections reflecting the converging evidence streams.

### 3.1. Biological and Mechanistic Evidence for a J-Shaped Response

#### 3.1.1. Hormesis and Biphasic Dose–Response

The classical pharmacological model, which has underpinned understanding of drug action for nearly a century, assumes that a drug’s effect is proportional to the fraction of receptors it occupies. The term receptor refers to a cellular macromolecule to which a drug binds to initiate its effects. In the simplest cases, the relationship between a drug’s dose and its effect is described by the familiar sigmoidal curve obtained when the magnitude of the effect is plotted against the logarithm of the drug dose. This sigmoidal curve has three features: a threshold, a slope, and a maximal asymptote [22]. The model assumes that the dose has no effect until a threshold is reached and that a maximal response occurs when further increases in the dose have no effect. However, in this classical concept, exceptions are quite common.

Probably the most intriguing exception to the standard pharmacological model is the observation that different compounds, including many clinically relevant drugs, exhibit opposite effects at different doses. The phenomenon in which small doses produce effects opposite to those of larger doses is commonly called hormesis. The hormetic dose–response relationship can be described as either an inverted U-shaped or a J-shaped curve, depending on the endpoint measured. For example, for CVD incidence, results are typically represented as J-shaped dose–response curves [23,24]. In the context of human nutrition, the hormetic biphasic effects of alcohol consumption on human health have been particularly well studied. The vast majority of epidemiological studies repeatedly demonstrate a J-shaped, biphasic dose–response association between alcohol consumption and various diseases and total mortality.

In the case of wine, the two principal constituents considered responsible for the majority of wine’s biological effects are alcohol (ethanol) and (poly)phenolic compounds. Indeed, these constituents have been attributed numerous distinctive biological effects that may confer health benefits. Moreover, both ethanol and the phenolic compounds in wine display hormetic biphasic dose–response relationships across multiple biological systems, mirroring the epidemiological J-curve [25,26,27,28].

#### 3.1.2. Polyphenols and Antioxidant Effects

Wine, particularly red wine, contains a diverse range of phenolic compounds—such as resveratrol, quercetin, catechins, anthocyanins, and procyanidins—that exert pronounced antioxidant effects. These compounds have been shown to act as chemical antioxidants, which may scavenge free radicals and chelate transition metals, such as iron, that are implicated in biochemical reactions associated with free radical production. While these mechanisms are relevant in cell-free systems, basic physiological and chemical kinetic considerations rule out direct free radical scavenging by polyphenols as a significant component of antioxidant defence in cells [29,30].

Instead, the antioxidant activity of phenolic compounds is mainly due to their ability to activate the cellular antioxidant defence system by mimicking the effects of endogenously produced oxidants. Namely, (poly)phenols such as resveratrol may act as pro-oxidants under physiologically relevant conditions. Pro-oxidative products of resveratrol activate multiple redox pathways in the cell. The cellular response to this stimulation is increased production of antioxidant enzymes and nucleophiles, thereby increasing the cell’s capacity to resist and survive more severe stress. Importantly, the adaptive mechanisms described are dose-dependent and exhibit a biphasic hormetic dose–response pattern. At low doses of phenolic compounds, cellular endogenous defence systems are stimulated, whereas at higher concentrations, these compounds lose efficacy and may become toxic [27,28,30,31]. These adaptive responses likely contribute to the biological effects of wine that differ from those of an equivalent amount of alcohol.

#### 3.1.3. Endothelial Function and Nitric Oxide Bioavailability

Moderate wine consumption improves endothelial function largely by increasing nitric oxide (NO) bioavailability. This occurs through increased endothelial nitric oxide synthase (eNOS) activity, thereby promoting vascular relaxation, reducing vasoconstriction, and enhancing flow-mediated dilation. Numerous controlled human studies show that both acute and chronic wine consumption yield greater improvements in endothelial responsiveness than ethanol alone, suggesting that wine’s phenolic constituents contribute substantially to these vascular effects [32,33,34,35,36].

#### 3.1.4. Anti-Inflammatory Actions

Moderate wine consumption has been shown to reduce systemic inflammation by lowering C-reactive protein, interleukin-6, tumour necrosis factor-α, and endothelial adhesion molecules, including ICAM-1 and VCAM-1 [37,38,39]. These effects align with reduced NF-κB signalling activity, which regulates immune, inflammatory, stress, proliferative, and apoptotic responses to multiple stimuli [40,41,42,43]. The cumulative anti-inflammatory effects contribute to reduced atherosclerotic progression and lower cardiometabolic risk.

#### 3.1.5. Platelet Function and Coagulation

Moderate wine consumption also affects haemostatic balance. Evidence indicates that wine reduces platelet aggregation, lowers fibrinogen levels, modulates Factor VII activity, and promotes endogenous fibrinolysis [44,45,46,47]. Taken together, these effects reduce the risk of thrombosis, complementing the vascular and metabolic benefits described above.

#### 3.1.6. Glucose Metabolism and Insulin Sensitivity

Moderate wine consumption improves glucose metabolism by enhancing insulin sensitivity, lowering fasting glucose concentrations, and reducing the risk of type 2 diabetes [48,49,50,51,52,53]. Large prospective cohort studies likewise report that moderate wine consumption is associated with a 15–30% lower incidence of type 2 diabetes than abstaining, with wine generally showing a stronger inverse association than beer or spirits [54,55,56,57,58]. These metabolic effects contribute to the reduction in all-cause and cardiovascular mortality observed with moderate wine consumption.

### 3.2. Evidence from Animal and Cellular Studies

#### 3.2.1. Differential Effects of Wine vs. Ethanol

Animal studies provide further evidence that wine exerts protective effects beyond those of ethanol alone. In a recent study examining the effects of four weeks of moderate consumption of white wine and ethanol on the survival of rats subjected to surgically induced myocardial infarction, the survival rate was highest in the wine-drinking group (72.2%) compared with the ethanol group (64.7%) and water-only controls (47.8%). Analysing ethanol consumption and survival outcomes, it was observed that surviving rats consumed significantly less ethanol than those that died, indicating an upper limit to ethanol’s protective effects. In contrast, in the wine group, no such upper limit was observed. This finding suggests that the non-ethanolic constituents of wine confer additional cardioprotective effects, enhancing myocardial resilience and reducing post-ischaemic injury [59]. In a subsequent immunohistochemical study examining the inflammatory aspects of post-infarction healing in the hearts of the same experimental animals, it was again confirmed that wine showed more favourable effects compared with ethanol alone and water controls.

Wine consumption was associated with the most beneficial ‘reparative’ environment in infarct-affected cardiac tissues. The ratio of anti-inflammatory (M2) to pro-inflammatory (M1) macrophages in the post-infarction myocardium was highest in the wine group, as indicated by the highest CD163/CD68 macrophage antigen ratio. This ratio decreased with increasing ethanol intake but remained significantly higher in animals receiving ethanol as wine.

Increased CD163/CD68 ratios at lower levels of ethanol consumption and decreased ratios at higher levels align with the biphasic J-shaped relationship observed in epidemiological studies of the association between alcohol consumption and human health.

In this study, the relative ranking of CD163/CD68 ratios across the experimental groups closely mirrored the animals’ survival rates after surgically induced myocardial infarction. These findings suggest that non-alcoholic components of wine may contribute to effects beyond those of ethanol alone on post-infarction inflammation and healing, as evidenced by the higher survival rate among wine-consuming animals [60].

#### 3.2.2. Resveratrol Hormesis

Resveratrol is considered the most studied phenolic compound in wine and is probably one of the most studied natural compounds overall. Although its concentration in wine is relatively low and varies compared with that of some other phenolic compounds [61], it has attracted the most scientific attention because of its early association with the ‘French paradox’, the observation that moderate consumption of red wine may be protectively associated with CHD [62].

Resveratrol has been associated with numerous beneficial biological effects, including antioxidant, anti-inflammatory, immunomodulatory, cardioprotective, and anticancer properties. These effects are mediated through multiple mechanisms and have been demonstrated in various *in vitro* and *in vivo* models [63]. Although many experimental studies have used resveratrol concentrations exceeding those achievable through moderate wine consumption, the rapid metabolism of resveratrol and the potential biological activity of its metabolites mean that direct comparisons between experimental doses and dietary exposure should be interpreted with caution [64,65]. However, as with the antioxidant properties of phenolic compounds, resveratrol’s effects also exhibit a hormetic-like biphasic dose–response, in which low doses are typically associated with beneficial effects, while high doses are usually toxic [66,67,68].

In this context, it is noteworthy that there is an imbalance between the plethora of publications describing the beneficial effects of resveratrol and the relatively few reports investigating its potential toxicity. The limited data on resveratrol’s toxic effects should inform the design of interventional studies involving resveratrol supplementation. Determining the optimal dosage of resveratrol to maximise health benefits without increasing the risk of toxicity remains an area of ongoing research [69].

#### 3.2.3. HDL Subspecies and Lipoprotein Function

Beyond traditional lipid effects, recent evidence shows that alcoholic beverages, particularly wine, favourably modify HDL subspecies composition [70]. HDL particles lacking apolipoprotein C3 (apoC3) are strongly cardioprotective, whereas those containing apoC3 are associated with increased CHD risk. Moderate wine consumption increases the proportion of HDL particles without apoC3 while decreasing the proportion containing apoC3, thereby shifting the HDL pool towards a more protective phenotype. This mechanistic refinement offers a compelling explanation for the well-documented HDL-mediated benefits of moderate wine consumption and addresses limitations of earlier Mendelian randomisation studies that focused solely on HDL-C mass rather than functionally distinct HDL subspecies [71]. In addition, lipoprotein (a), an independent lipid risk factor for atherosclerosis and CVD [72], has been shown to be reduced by wine-derived phenolic compounds [73,74].

#### 3.2.4. ApoB and ApoB/ApoA1 Ratio

Recent Mendelian randomisation analyses have identified apolipoprotein B (apoB), which reflects the total number of atherogenic lipoprotein particles, as a key causal factor in coronary risk [75]. In contrast, apolipoprotein A-I (apoA-I) is the principal structural protein of high-density lipoprotein (HDL) particles and the primary mediator of reverse cholesterol transport. Together, the apoB/apoA-I ratio reflects the balance between atherogenic and anti-atherogenic lipoproteins and has emerged as a strong predictor of cardiovascular risk [76]. Randomised wine intervention studies have historically focused on HDL-related apolipoproteins rather than apoB. These trials consistently demonstrate increases in apoA-I (and occasionally apoA-II), whereas apoB has rarely been included in the measurement outcomes. The landmark randomised crossover study by Goldberg et al. [77] measured apoB alongside apoA-I and apoA-II and demonstrated significant increases in apoA-I and the apoA-I:apoB ratio, despite no change in apoB concentrations. This indicates that the improved ratio was driven primarily by increased apoA-I rather than reduced apoB. Similarly, observational studies have reported lower apoB/apoA-I ratios among individuals with low-to-moderate alcohol intake, an association that appears to be mediated predominantly by higher apoA-I concentrations rather than lower apoB [78,79]. In total, these findings support the biological plausibility that moderate wine consumption favourably influences lipoprotein metabolism, although direct evidence that wine lowers apoB remains limited. Therefore, contemporary randomised trials incorporating apoB and the apoB/apoA-I ratio as prespecified endpoints are warranted.

#### 3.2.5. Thrombosis, Fibrinolysis, and Platelet Function

Moderate wine consumption reduces platelet aggregability, enhances endothelial fibrinolysis, and favours a more balanced coagulation. Experimental studies demonstrate that red wine inhibits platelet aggregation more effectively than ethanol alone [80,81,82,83,84] and prevents the rebound hyperaggregability observed after alcohol withdrawal [85]. Recent studies also show that moderate wine consumption improves the activity of PAF-metabolising enzymes, independently of ethanol, and reduces platelet aggregation, probably through mechanisms distinct from those of ethanol [86]. Low-dose, regular wine exposure also upregulates endothelial tissue plasminogen activator (tPA) and urokinase pathways, thereby enhancing endogenous fibrinolysis [87,88]. These mechanisms provide a coherent biological explanation for the repeatedly observed reduced risk of acute myocardial infarction among moderate drinkers.

#### 3.2.6. Integrating Biological Mechanisms with Epidemiological Evidence

The available mechanistic evidence provides a coherent biological framework for explaining the epidemiological associations observed with moderate wine consumption. Rather than acting through a single pathway, wine appears to influence multiple interconnected processes that underpin cardiometabolic health. Experimental studies indicate that ethanol itself exhibits concentration-dependent (hormetic) effects on endothelial function, vascular remodelling, and inflammatory signalling, with low concentrations generally promoting adaptive vascular responses that differ from those at higher concentrations [89]. Wine-derived polyphenols may further enhance endothelial nitric oxide bioavailability, reduce oxidative stress and vascular inflammation, improve lipoprotein function, and inhibit platelet activation. These complementary mechanisms provide biological plausibility for the lower incidence of cardiovascular disease, reduced all-cause mortality, and improved metabolic outcomes observed in prospective cohort studies. Although residual confounding and healthy-user effects cannot be excluded, the convergence of mechanistic, intervention, and epidemiological evidence strengthens the biological plausibility of the observed J-shaped relationship.

### 3.3. Epidemiological Evidence: J-Shaped Associations

#### 3.3.1. Total Mortality

Numerous prospective cohort studies consistently show that light-to-moderate wine consumption, typically corresponding to average intakes of 5–15 g of ethanol/day for women and 10–20 g/day for men, is associated with the lowest risk of all-cause mortality [90]. Although the precise nadir of the J-shaped association varies across populations by age, sex, drinking pattern, baseline health status, and study methodology, these intake ranges are broadly comparable to those reported in major prospective cohort studies and dose–response meta-analyses across European, North American, and Australasian populations [6,7,9,90,91]. Mortality risk rises among heavy drinkers and is generally similar to, or slightly higher than, that of abstainers, compared with moderate wine drinkers [91,92,93,94]. Notably, the inverse association is stronger for wine than for beer or spirits, indicating a beverage-specific effect supported by epidemiological and mechanistic findings.

#### 3.3.2. Cardiovascular Disease (CVD)

Moderate wine consumption is associated with substantial reductions in cardiovascular risk. Multiple studies show that individuals who drink moderate amounts of wine have lower rates of coronary heart disease, stroke, heart failure, and overall cardiovascular mortality [95,96,97,98,99,100,101,102,103,104,105]. These epidemiological findings align with mechanistic evidence of improved endothelial function, reduced inflammation, antioxidant activity, and favourable lipid modulation among wine consumers.

#### 3.3.3. Diabetes and Metabolic Outcomes

Moderate wine consumption is consistently associated with improved metabolic health. Studies show that compared with abstainers or heavy drinkers, moderate wine drinkers have lower rates of type 2 diabetes, better insulin sensitivity, and more favourable glucose levels [57,106]. These findings help explain the decline in cardiometabolic mortality observed among moderate drinkers.

#### 3.3.4. Neurodegenerative Disorders

Several cohort studies demonstrate that moderate wine consumption is associated with a lower risk of cognitive decline, dementia, and Alzheimer’s disease [13,107,108,109]. Large prospective studies and systematic reviews report a 20–45% lower risk of cognitive decline or Alzheimer’s disease among light-to-moderate wine consumers, with proposed mechanisms including attenuation of vascular inflammation, reduced oxidative stress, improved endothelial and microvascular function, and neuroprotective effects of wine polyphenols such as resveratrol and flavonoids [10].

#### 3.3.5. Evidence Across Subgroups

Multiple cohort studies show that the J-shaped association persists across diverse subgroups, including older adults, patients with type 2 diabetes, individuals with hypertension, and those with established cardiovascular disease. For example, among 99,654 adults aged 55–74 years, lifetime alcohol consumption showed a clear J-shaped association with all-cause and cardiovascular mortality, with elevated risk among both abstainers and heavier consumers [110]. In a pooled analysis of 333,247 US adults followed for over a decade, light (HR 0.79) and moderate (HR 0.78) drinkers had significantly lower all-cause mortality than lifetime abstainers, whereas heavy drinking was associated with higher mortality (HR 1.11) [8]. These findings suggest that the protective association is not confined to specific age or health groups and persists after extensive adjustment for lifestyle factors and comorbidity.

#### 3.3.6. Beverage-Specific Comparisons

Mechanistic, intervention, and prospective cohort studies generally suggest that moderate wine consumption is associated with more favourable cardiometabolic outcomes than beer or spirits, although the magnitude of any beverage-specific difference remains uncertain. Early comparative evidence also cautioned against assuming that the apparent advantage of wine was universal. In a systematic review of ecological, case–control, and prospective cohort studies, Rimm et al. (1996) found inverse associations between coronary heart disease and moderate consumption of wine, beer, and spirits, concluding that a substantial proportion of the observed cardiovascular benefit was attributable to alcohol itself, while any additional benefit from beverage-specific components was likely modest or population-specific [111]. While ethanol contributes to several biological effects common to all alcoholic beverages, the polyphenolic matrix of wine may support improved endothelial function, reduce oxidative stress and inflammation, enhance lipoprotein function, and reduce platelet aggregation [39,112,113,114,115,116]. Nevertheless, direct comparative intervention studies across beverage types remain limited, and residual confounding by dietary and lifestyle factors should continue to be considered when interpreting beverage-specific associations.

Accordingly, beverage type is an important consideration when interpreting epidemiological findings, and analyses that treat all alcoholic beverages as interchangeable may overlook potentially relevant biological and behavioural differences.

### 3.4. Cancer Outcomes and Contextual Risk Interpretation

Public health often treats cancer as a single disease, yet it comprises multiple diseases. The relationship between alcohol consumption and cancer risk is complex and varies by cancer type. For some cancers, including oral and oesophageal cancers, risk increases linearly with higher levels of alcohol intake, whereas for others, such as breast and colorectal cancers, modest associations have been reported at lower levels of consumption. However, multiple interacting factors influence these associations, including genetic susceptibility, hormonal status, body mass index, smoking, diet, and physical activity [117].

The association between alcohol and cancer varies by anatomical site. The strongest and most consistent evidence concerns breast cancer, for which risk appears to increase approximately linearly with alcohol intake, even at relatively low levels of consumption, although the magnitude of the association varies by population and baseline risk [118]. Associations have also been reported for cancers of the oral cavity, pharynx, larynx, oesophagus, colorectum, and liver, particularly at higher levels of alcohol consumption. By contrast, evidence for several other cancer sites is inconsistent or limited, and some observational studies have reported inverse associations for selected outcomes, although these findings remain subject to confounding and should be interpreted cautiously. Consequently, interpretation of alcohol-related cancer risk requires consideration of site-specific differences, dose, drinking pattern, competing causes of morbidity and mortality, and both absolute and relative risk.

At low levels of consumption, absolute increases in cancer risk are generally small, though the magnitude varies by cancer site, age, sex, and baseline risk. Importantly, alcohol contributes a relatively modest proportion of the total cancer burden, accounting for approximately 4–5% of incident cancers, a figure that has remained stable over time [119]. Among modifiable risk factors, alcohol ranks third after tobacco smoking and excess body weight in its contribution to the overall cancer burden [120].

Interpreting alcohol-related cancer risk is further complicated by limitations in current modelling methods, which generally do not differentiate between beverage types or drinking patterns. As a result, the potential biological effects of wine’s phenolic compounds, which have been shown to influence pathways involved in oxidative stress, inflammation, and carcinogenesis, are not accounted for. Additionally, regular moderate consumption differs significantly from episodic heavy drinking, which is more strongly associated with adverse outcomes.

The dietary context is also important. Adhering to dietary patterns such as the Mediterranean diet, which includes moderate wine consumption, has consistently been associated with a lower overall cancer risk [121]. Meta-analytic evidence suggests that moderate wine intake within this dietary context has not been consistently linked to increased overall cancer risk and commonly occurs alongside other features of a higher-quality diet, including greater consumption of fruits and vegetables.

Overall, these findings suggest that the relationship between moderate wine consumption and cancer varies by cancer site [114,122]. The strongest evidence for an increased risk at low levels of alcohol consumption concerns breast cancer, whereas risks for several other alcohol-related cancers are more evident at higher levels of consumption. Importantly, the absolute increase in cancer risk associated with low levels of wine consumption appears modest and should be considered alongside competing health outcomes, individual baseline risk, and the overall balance of evidence discussed below.

### 3.5. International Alcohol Guidelines and Policy Context

Cardiovascular societies and national health authorities issue alcohol consumption guidelines to reduce overall health risk, including CVD risk. In the absence of large-scale, randomised, controlled trials, these recommendations are necessarily informed by observational evidence and, latterly, Mendelian randomisation evidence. Across jurisdictions, there is broad consensus that alcohol consumption should not be initiated for health benefits and that abstinence is appropriate for individuals with contraindications. However, given the widespread global prevalence of alcohol consumption, most guidelines adopt harm reduction approaches based on defined intake thresholds.

Notably, recommended limits vary across regions. The most recent Canadian guidelines, published in 2023, recommend limiting consumption to no more than two drinks per week for ‘low risk’ drinking, making it one of the strictest thresholds globally [123]. These guidelines are largely based on GBD 2020 estimates, which utilise a linear, cancer-focused risk model and do not account for protective cardiometabolic effects. The 2020 Australian guidelines lowered the recommended upper limit for adults to no more than 100 g of ethanol per week, applied equally to men and women [18]. This revision relied heavily on modelling from the Sheffield Alcohol Research Group, which developed comparative risk models that estimate alcohol-attributable harm by integrating epidemiological risk functions across multiple diseases and levels of alcohol consumption. The 2016 U.K. guidelines also used this approach and set an upper limit of 112 g of ethanol per week for both sexes [124].

In contrast, the U.S. guidelines for primary prevention of cardiovascular disease propose sex-specific thresholds of ≤2 standard drinks per day for men and ≤1 drink per day for women [125]. European guidance, however, generally adopts lower, non-sex-specific limits, typically around ≤100 g per week and ≤15 g per day, although national recommendations vary. These discrepancies highlight a lack of uniformity in how alcohol-related risk is interpreted and translated into policy.

Despite general agreement on limiting alcohol intake, recent guideline revisions have moved towards more restrictive thresholds. These changes reflect not only increased attention to alcohol-related cancer risk but also differences in the methods used to estimate harm. Understanding these methodological differences is therefore important for interpreting current policy directions.

### 3.6. Methodological Drivers of Policy Change

While international guidelines increasingly converge on lower recommended thresholds, these changes are driven not only by new biological evidence but also by evolving risk estimation methods. The principal methodological factors shaping recent guideline development are summarised in Table 2 and discussed below.

Recent shifts in alcohol guidelines, particularly those of the U.K., Australia, and Canada [18,123,124,125], have been influenced not only by new epidemiological evidence but also by changes in the methodological frameworks used to interpret it. Several methodological decisions are particularly important. These include the selection of disease endpoints in comparative risk models, the treatment of alcoholic beverages as a homogeneous exposure, the emphasis on relative rather than absolute risk, assumptions about acceptable lifetime risk thresholds, and the extent to which models account for age, drinking pattern, and competing causes of mortality. Together, these decisions can materially influence the estimated risk at low levels of consumption and, therefore, the drinking thresholds recommended in national guidelines. Recent commentaries have argued that many apparent changes in alcohol policy reflect differences in the interpretation of evidence rather than fundamental changes in the underlying biological or epidemiological evidence [9,123,126,127,128].

Additional methodological factors contribute to divergence from established epidemiological evidence. These include limited age stratification, despite substantial variation in competing risks across the life course, as illustrated by comparative risk models that rely on population-wide estimates rather than age-specific risk–benefit profiles [18,124]; insufficient consideration of drinking patterns, in which episodic heavy consumption is often analysed together with regular moderate intake, despite evidence that these patterns have markedly different biological and clinical consequences [7,40,125]; and treating all alcoholic beverages as homogeneous exposures expressed solely as grams of ethanol, thereby overlooking beverage-specific differences in composition, drinking context, and the potential contribution of wine polyphenols [39,123,127]. Furthermore, some comparative risk models prioritise specific alcohol-attributable disease endpoints while excluding cardioprotective outcomes and all-cause mortality from the overall assessment, thereby altering the estimated balance of risks and benefits at low levels of consumption [18,124,126]. These exclusions can alter the shape and interpretation of the dose–risk relationship by assigning less weight to the descending limb of the J-shaped association, thereby favouring a more monotonic interpretation of risk. Overall, these methodological choices may lead to higher estimated risks at low levels of consumption and partially explain the trend towards more restrictive drinking recommendations.

A more balanced, biologically informed framework that incorporates absolute risk, age-specific effects, drinking patterns, beverage type, and the full spectrum of health outcomes is therefore essential to developing coherent, evidence-based alcohol policy.

**Table 2:** Principal methodological considerations influencing the interpretation of evidence in recent alcohol guideline development.

Methodological Factor	Illustrative Example	Potential Influence on Guideline Recommendations	Representative Examples
Disease endpoint selection	Greater emphasis on alcohol-related cancers, with less consideration of cardioprotective outcomes or all-cause mortality	May produce higher estimated risks at low levels of consumption and attenuate the J-shaped association	GBD 2020; NHMRC 2020; CCSA 2023 [18,20,127]
Treatment of alcoholic beverages	Wine, beer, and spirits analysed together as grams of ethanol	May not adequately capture beverage-specific effects, drinking context, or wine polyphenol effects	GBD 2020; Shield et al. 2024 [127,129]
Relative vs. absolute risk	Small relative increases reported without corresponding absolute risk	May overemphasise the public health significance of very small absolute increases in risk	NHMRC 2020; Conigrave et al. 2021 [18,130]
Age stratification and competing risks	Limited consideration of differences between younger and older adults	May not reflect the changing balance between cardiovascular disease and other alcohol-related harms over the life course	GBD 2020; Wood et al. 2018 [9,127]
Drinking pattern	Regular moderate consumption analysed together with episodic heavy drinking	May obscure important differences in biological effects and health outcomes	Ronksley et al. 2011; Conigrave et al. 2021 [6,130]
Acceptable risk thresholds	Different countries adopt different thresholds for lifetime risk	Can produce substantially different drinking recommendations despite similar underlying evidence	UK Chief Medical Officers 2016; NHMRC 2020; CCSA 2023 [18,19,20]

## 4. Discussion

The evidence reviewed supports the biological plausibility and epidemiological consistency of a reported J-shaped association between moderate wine consumption and reduced all-cause mortality, largely driven by cardiometabolic outcomes. However, interpreting this association remains the subject of ongoing scientific debate, as different study designs provide complementary but incomplete perspectives on causality. Mechanistic, intervention, observational, Mendelian randomisation, and comparative risk-modelling studies each have distinct strengths and limitations that should be considered collectively rather than in isolation. This distinction between stable evidence and evolving methodology is central to interpreting the divergence between long-standing epidemiological findings and more recent assertions that “no safe level of alcohol exists.” Overall, the evidence indicates that heavy or irregular drinking is harmful, whereas low-to-moderate wine consumption is associated with more favourable cardiovascular and metabolic outcomes, particularly among middle-aged and older adults.

### 4.1. Biological Plausibility of Wine-Specific Cardioprotection

Moderate wine consumption is biologically plausible as being cardioprotective, owing to its phenolic matrix, which influences oxidative stress, inflammation, endothelial function, glucose metabolism, and platelet activity, thereby supporting vascular homeostasis and potentially slowing atherosclerotic processes. A hormetic dose–response, consistent with the J-shaped relationship, indicates that low doses activate protective cellular pathways, whereas higher doses may be harmful. Experimental evidence also indicates that wine provides greater cardioprotection than ethanol alone, probably due to its phenolic components.

### 4.2. Epidemiological Consistency of the J-Shaped Curve

Large prospective cohort studies report that moderate wine consumption is linked to the lowest all-cause mortality, with risk rising among both abstainers and heavier drinkers. Wine consumption has also been associated with lower risks of coronary heart disease, type 2 diabetes, cognitive decline, and overall cardiovascular mortality. These associations vary by dose, drinking pattern, beverage type, age, and underlying health status, factors that are not uniformly incorporated into recent comparative risk models and guideline frameworks.

Interpretation of these findings, however, requires caution. Observational studies cannot fully establish causality, and moderate wine consumers often differ from abstainers and heavier drinkers in dietary quality, physical activity, smoking behaviour, socioeconomic status, healthcare utilisation, and other health-related behaviours. Although most contemporary cohort studies adjust for these factors, residual confounding and healthy-user bias cannot be entirely ruled out. Similarly, reverse causation and former-drinker bias may contribute to the elevated risks observed among abstaining groups if individuals reduce or cease alcohol consumption because of declining health.

At the same time, it would be wrong to conclude that the reported J-shaped association is entirely explained by these biases. The consistency of findings across numerous prospective cohorts, together with evidence from mechanistic studies, human intervention trials, and beverage-specific analyses, supports the biological plausibility of favourable associations with moderate wine consumption. The remaining challenge, therefore, is not merely to determine whether the association exists but to quantify more precisely the relative contributions of biological mechanisms, drinking patterns, beverage composition, lifestyle factors, residual confounding, and individual biological variability.

### 4.3. Cancer Risk in a Risk–Benefit Framework

Although alcohol consumption is causally linked to several cancers, the absolute risk increase at low levels of intake is small and strongly modified by age, baseline risk, and behavioural and environmental factors. In older populations, where cardiovascular disease accounts for a larger share of the disease burden, potential cardiometabolic benefits may carry greater weight in the overall risk–benefit assessment.

Conversely, in younger adults, who have a low absolute risk of cardiovascular disease but a higher likelihood of alcohol-related injuries, violence, or other acute harms, the balance of risks and benefits is likely to differ [9,127]. Consequently, population-level recommendations for younger adults appropriately place greater emphasis on avoiding alcohol-related harm, whereas potential cardiometabolic benefits become more relevant in middle-aged and older populations with higher baseline cardiovascular risk.

Many modelling approaches do not account for beverage type, drinking pattern, or dietary context, potentially overstating the risk associated with moderate wine consumption. Accordingly, cancer risk should be interpreted within a broader risk–benefit framework rather than in isolation.

### 4.4. How Modelling Choices Altered Guideline Outcomes

The implications of these modelling choices are significant, as they shape how epidemiological evidence is translated into population-level recommendations. Although comparative risk models offer a structured way to integrate multiple disease outcomes, differences in outcome selection, exposure assumptions, disease weighting, and acceptable risk thresholds can yield substantially different estimates of net risk at low levels of consumption. Thus, differences between successive guideline recommendations may reflect not only evolving evidence but also shifts in methodological frameworks and public health priorities.

### 4.5. Public Understanding and Behavioural Impact

Despite these policy shifts, evidence that revisions to drinking guidelines alone have produced substantial changes in population drinking behaviour remains limited. Evaluations of the revised U.K. low-risk drinking guidelines found that although awareness of the revision increased after its introduction, there was little evidence of a sustained reduction in alcohol consumption attributable to the guideline change itself [15]. Broader long-term declines in alcohol consumption observed in several countries are likely to reflect multiple interacting influences, including changing social norms, demographic shifts, pricing policies, health awareness, and other public health interventions, rather than the guideline revisions alone. Furthermore, surveys indicate that public knowledge of the specific drinking recommendations remains relatively poor, despite increased awareness of the guidelines [16]. These findings suggest that effective alcohol policy requires not only evidence-based guideline development but also clear, credible, and accessible risk communication. Public messaging perceived as inconsistent with the broader epidemiological and biological evidence may undermine public trust and engagement.

### 4.6. Implications for Nutritional and Public Health Recommendations

Overall, the evidence indicates that moderate wine consumption can be compatible with a healthy dietary pattern when consumed regularly with meals and without binge drinking. The net effect is context-dependent and varies with age, baseline risk, beverage type, and drinking pattern. The balance of evidence suggests that any net cardiometabolic benefit is most likely to be observed among middle-aged and older adults with higher baseline cardiovascular risk. This balance is likely to differ among younger adults, in whom acute alcohol-related harms contribute more substantially to overall risk. These findings align with dietary models such as the Mediterranean diet.

However, it is important not to assume that findings from Mediterranean populations are directly generalisable to all settings. Much of the evidence supporting favourable associations with moderate wine consumption comes from Mediterranean populations, where wine is typically consumed with meals as part of an overall dietary pattern rich in plant foods, olive oil, and fish and accompanied by distinctive lifestyle behaviours [115,131,132]. Although similar associations have been reported in cohorts from North America, Northern Europe, Australia, and other regions [3,4,5,6,7,8], differences in dietary composition, drinking culture, beverage preferences, and alcohol consumption patterns may influence the magnitude and interpretation of these associations. Consequently, any potential health benefits of wine are most consistently observed when wine is consumed in moderation, with meals, and as part of an overall healthy dietary and lifestyle pattern, rather than as an isolated exposure [116].

Accordingly, public health strategies should prioritise reducing heavy and episodic drinking while providing nuanced guidance that reflects the complexity of risk, thereby avoiding overgeneralisation and maintaining credibility.

Operationalising a more balanced evidence framework need not entail different drinking limits but rather greater transparency in how evidence is integrated and communicated. Future guideline development could more explicitly communicate both absolute and relative risk, distinguish drinking patterns from average alcohol intake, recognise that alcohol-related risk varies across the life course according to baseline health status and competing causes of morbidity and mortality, and transparently acknowledge where evidence differs by beverage type or dietary context. Such an approach would better reflect the complexity of the available evidence, improve public understanding of alcohol-related risk, maintain a strong emphasis on reducing alcohol-related harm, and support evidence-informed, context-sensitive public health guidance [129,130].

At the same time, public health guidance must balance scientific nuance with the need for clear, practical communication. A hybrid approach that combines simple population-level recommendations with a transparent explanation of how risk varies by age, drinking pattern, baseline health status, and, where evidence supports it, beverage type may better promote public understanding while maintaining trust and supporting informed decision-making [129].

### 4.7. Future Directions: Towards Precision Nutrition and Precision Prevention

An important implication of this review is that future research should move beyond estimating average population effects and instead investigate heterogeneity in the relationship between wine consumption and health outcomes. Cardiovascular and neurodegenerative outcomes are influenced not only by alcohol exposure but also by age, sex, cardiometabolic status, genetic susceptibility, dietary patterns, socioeconomic circumstances, comorbidities, medication use, and other behavioural and environmental factors. Consequently, the risks and potential benefits of moderate wine consumption are unlikely to be uniform across populations.

Emerging concepts in precision nutrition and precision prevention offer an opportunity to better understand these differences. Future research should integrate large prospective epidemiological cohorts with nutritional data, clinical registries, biomarker profiling, and, where appropriate, genomic information to identify subgroups that may respond differently to moderate wine consumption. Such studies should also assess whether differences in response are modified by sex, ancestry, and genetic polymorphisms affecting alcohol metabolism (including ADH and ALDH variants) [133,134], as well as other determinants of susceptibility that may influence both beneficial and adverse health outcomes. In addition, well-designed randomised controlled trials are needed to examine moderate wine consumption in realistic dietary settings, particularly among middle-aged and older adults at elevated cardiometabolic risk. Although long-term randomisation of alcohol intake poses practical and ethical challenges, shorter-term intervention studies using validated intermediate endpoints could provide useful mechanistic and clinical evidence. Long-term prospective cohort studies incorporating genetic, metabolomic, dietary, and lifestyle data could then help clarify the independent and interactive contributions of wine, drinking pattern, and overall dietary context to health outcomes. Such approaches may help distinguish the relative contributions of ethanol, wine-derived bioactive compounds, drinking pattern, Mediterranean dietary patterns, and residual confounding to observed health outcomes.

Rather than asking whether moderate wine consumption is universally beneficial or harmful, future studies should determine, based on individuals’ genetic, metabolic, demographic, and lifestyle characteristics, which individuals, if any, may derive cardiovascular or neurocognitive benefits, experience no measurable effect, or face increased risk. Such evidence could ultimately support more personalised public health recommendations while recognising that population-level guidance remains necessary to protect overall public health.

### 4.8. Limitations of the Evidence

This review acknowledges several important limitations. Evidence on moderate wine consumption is derived predominantly from observational studies, which remain susceptible to residual confounding, reverse causation, healthy-user bias, exposure misclassification, under-reporting of alcohol intake, and heterogeneity in drinking patterns. Conversely, mechanistic and intervention studies typically have shorter durations and rely on surrogate endpoints that cannot fully establish long-term clinical outcomes. Mendelian randomisation studies and comparative risk models depend on assumptions that may not fully capture beverage-specific behaviours or drinking context. No single study design is sufficient to resolve these uncertainties. Accordingly, conclusions are drawn from complementary evidence across multiple methodological approaches.

The ongoing debate about moderate wine consumption should not, therefore, be framed as a choice between accepting or rejecting the J-shaped association. Rather, it reflects the challenge of integrating evidence from fundamentally different methodological approaches, each addressing distinct aspects of causality and risk. Future advances are likely to stem from combining mechanistic, clinical, epidemiological, and precision health research, rather than privileging any single methodology.

## 5. Conclusions

Mechanistic, epidemiological, and clinical evidence support the biological plausibility and epidemiological consistency of a reported J-shaped association between moderate wine consumption and several important health outcomes, particularly cardiovascular disease and all-cause mortality. Experimental studies demonstrate mechanisms through which both ethanol and wine-derived phenolic compounds may influence oxidative stress, inflammation, endothelial function, and metabolic regulation. However, observational evidence remains subject to residual confounding, healthy-user bias, reverse causation, and other limitations that preclude definitive causal inference. Accordingly, the available evidence should be interpreted by integrating complementary findings from mechanistic studies, clinical investigations, epidemiological research, Mendelian randomisation studies, and comparative risk modelling.

The divergence between earlier evidence syntheses and more recent alcohol guidelines therefore likely reflects both evolving methodological approaches to risk assessment and ongoing uncertainty about the causal interpretation of observational evidence. Assumptions about outcome selection, overall mortality, cardioprotective effects, beverage type, drinking pattern, and comparative risk modelling can substantially influence estimates of population risk and the resulting recommendations.

Overall, the evidence suggests that moderate, regular wine consumption with meals may be compatible with a healthy dietary pattern for some adults, particularly in mid- to later life, although this relationship is unlikely to be uniform across populations. Future research should increasingly adopt precision nutrition and precision prevention approaches, integrating prospective epidemiological, clinical, dietary, biomarker, and genetic data to identify population subgroups with distinct risk–benefit profiles.

## Figures and Tables

**Table 1:** PICOS framework and study eligibility criteria used for the narrative review.

PICOS Element	Inclusion Criteria
Population	Adults from general population and clinical cohorts
Intervention/Exposure	Wine consumption, wine polyphenols, moderate alcohol consumption where relevant, drinking patterns
Comparator	Abstainers; low, moderate, and high consumers; placebo/control; other beverage types
Outcomes	All-cause mortality, cardiovascular disease, diabetes, cancer, dementia, cognition, biomarkers, endothelial function, inflammation, oxidative stress, guideline methodology
Study design	Mechanistic studies, human intervention trials, prospective cohort studies, case–control studies, systematic reviews, meta-analyses, Mendelian randomisation studies, guideline documents

## Data Availability

No new data were created or analysed in this study. Data sharing is not applicable to this article.
